# Dry Grangrene

**Published:** 1842-05

**Authors:** 


DRY GANGRENE.

The letter from which we cut the above, has the following paragraph. In due time we shall publish the result of the case.

"I have a case of dry gangrene, subject 83 years old, remarkably temperate and robust, was never sick; the disease began in August, has extended nearly to the knee, and is now separating from the sound flesh; with a little assistance nature now may perform a cure. I think the leg may be removed soon; he has taken no medicine of any moment for three months, until lately, and is now on a tonic course."

